# Digital Health Care, Telemedicine, and Medicolegal Issues in Orthopedics: A Review

**DOI:** 10.3390/ijerph192315653

**Published:** 2022-11-25

**Authors:** Davide Ferorelli, Lorenzo Moretti, Marcello Benevento, Maurizio Mastrapasqua, Michele Telegrafo, Biagio Solarino, Alessandro Dell’Erba, Davide Bizzoca, Biagio Moretti

**Affiliations:** 1 Section of Legal Medicine, Department of Interdisciplinary Medicine, University of Bari, 70121 Bari, Italy; 2 Orthopedics and Trauma Unit, AOUC Policlinico di Bari, Department DiBraiN, University of Bari “Aldo Moro”, 70121 Bari, Italy

**Keywords:** tele-orthopedics, telemedicine, legal medicine, clinical risk management

## Abstract

The use of technologies in medicine has great potential to reduce the costs of health care services by making appropriate decisions that provide timely patient care. The evolution of telemedicine poses a series of clinical and medicolegal considerations. However, only a few articles have dealt with telemedicine and orthopedics. This review assesses the ethical and medicolegal issues related to tele-orthopedics. A systematic review was performed including papers published between 2017 and 2021 focusing on the main medicolegal and clinical-governance aspects of tele-orthopedics. Most of the articles were published during the COVID-19 pandemic, confirming the impetus that the pandemic has also given to the spread of telemedicine in the orthopedic field. The areas of interest dealt with in the scientific evidence, almost exclusively produced in the USA, Europe, the UK, and Canada, are quality, patient satisfaction, and safety. The impact of telemedicine in orthopedics has not yet been fully evaluated and studied in terms of the potential medicolegal concerns. Most of the authors performed qualitative studies with poor consistency. Authorizations and accreditations, protection of patient confidentiality, and professional responsibility are issues that will certainly soon emerge.

## 1. Introduction

Telemedicine is a term coined in the 1970s to define “healing at a distance” [1]. Over the years, the concept of telemedicine has been better framed in the use of information communications technology (ICT) to improve patient outcomes by increasing access to care and medical information.

In 1997, the World Health Organization (WHO) adopted the following description for telemedicine: “The delivery of health care services, where distance is a critical factor, by all health care professionals using information and communication technologies for the exchange of valid information for the diagnosis, treatment and prevention of diseases and injuries, research and evaluation, and for the continuing education of health care providers, all in the interests of advancing the health of individuals and their communities” [2].

The current conception of telemedicine relies on this definition, understood as a method of the relationship between medical staff and patients, set in different locations, which results in a significant benefit to the care process through the reduction in time and distance [3].

Telemedicine services can be classified into three categories. Specialized telemedicine encompasses remote medical services provided within a specific medical discipline in support of the components of a more traditional clinical examination [4].

Telemedicine services include televisit, a medical act in which the physician interacts with the patient at a distance [5]; teleconsultation, an indication of diagnosis or therapy without the physical presence of the patient that provides the use of video to facilitate remote interaction between health care practitioners and patients [6]; and health telecooperation, which represents the assistance provided by a health professional to another health professional [7].

The second category is represented by telehealth care, which refers to the use of ICT to deliver health care at a distance and to support patient self-management through remote monitoring [8].

The last category is teleassistance, a social-assistance system for the management of frail persons at home through alarms, the activation of emergency services, or support calls from a service center [9,10].

The exponential growth of telemedicine poses a series of medicolegal problems, especially in terms of professional liability. To the best of our knowledge, no specific regulation of such matter is in force in Europe and only a few authors have dealt with it.

Orthopedics represents one of the main fields for the application of telemedicine and is one of the disciplines most involved in litigation due to professional liability [11,12]. Hence, it is assumed that litigations will increase along with the widespread of tele-orthopedics. Just like many other fields of application of telemedicine, tele-orthopedics encompasses services for consultation and follow-up (televisit, teleconsultation), cooperation within professionals (telecooperation), and surgery (telesurgery). Despite this, to the best of our knowledge, no articles have systematically addressed tele-orthopedics-related medicolegal issues.

Considering the existing gap in the literature, the present review aims to assess the main medicolegal concern linked to tele-orthopedics.

## 2. Materials and Methods

A systematic review of MEDLINE, Embase, and Web of Science was performed according to the PRISMA guidelines. No review protocol regarding tele-orthopedics was found in the Cochrane Library and PROSPERO. Searches were performed from 6 to 20 January 2022.

### 2.1. Eligibility Criteria

The authors considered papers written in English, with full-text available, published between 2017 and 2021. The topic focused on the main medicolegal and clinical-governance issues related to tele-orthopedics. The authors included every paper assessing the issue with qualitative and quantitative outcomes.

### 2.2. Information Sources and Search

The full electronic search strategy slightly changed between the databases due to the different index systems:

1. MEDLINE: “(((telehealth) OR (telehealth) OR (teleorthopedics) OR (tele-orthopaedics) OR (telesurgery) OR (telesurgery)) AND (orthopaedics AND ((ethics) OR (liability) OR (quality) OR (risk) OR (informed consent) OR (satisfaction) OR (misdiagnosis)”;
2. Embase: “TITLE-ABS-KEY (((telehealth) OR (telehealth) OR (teleorthopedics) OR (tele-orthopaedics) OR (telesurgery) OR (telesurgery)) AND (orthopaedics) AND ((ethical) OR (liability) OR (quality) OR (risk) OR (informed AND consent) OR (satisfaction) OR (quality) OR (privacy) OR (misdiagnosis) OR (misdiagnosis))) AND (LIMIT-TO (PUBYEAR, 2021) OR LIMIT-TO (PUBYEAR, 2020) OR LIMIT-TO (PUBYEAR, 2019) OR LIMIT-TO (PUBYEAR, 2018) OR LIMIT-TO (PUBYEAR, 2017))”;
3. Web of Science: “(((telehealth) OR (telehealth) OR (teleorthopedics) OR (tele-orthopaedics) OR (telesurgery) OR telesurgery)) AND ((ethical) OR (liability) OR (quality) OR (risk) OR (informed consent) OR (satisfaction) OR (quality) OR (privacy) OR (misdiagnosis) OR (misdiagnosis)) AND (orthopaedics)))”.

### 2.3. Selection of Sources of Evidence and Data Charting

Titles and abstracts were screened independently in duplicate by a couple of reviews to determine whether the retrieved studies met the inclusion criteria outlined above. A data charting form was jointly developed by the authors and tested before the data extraction. The data extraction was independently performed in duplicate by two reviewers. A third reviewer was consulted if needed during both the screening and extraction processes.

### 2.4. Data Items and Synthesis of Results

The extracted data were:

- Authors’ affiliation;
- Year of publication;
- Type of paper (original research, review, other);
- Considered issues (ethics, medical liability, informed consent, safety, quality, clinical risk management, patient satisfaction, provider satisfaction, privacy, and misdiagnosis);
- Setting (teleconsultation, telesurgery);
- Medical intervention (diagnostics, therapy, follow-up, unspecified);
- Comparison with face-to-face orthopedics (yes, no);
- Main outcome (positive, negative).

The authors summarized the comparison between tele-orthopedics and face-to-face orthopedics as the “main outcome”. The “positive” main outcome was assigned to papers that presented tele-orthopedics as a suitable alternative to face-to-face orthopedics.

To synthesize the qualitative evidence, the authors chose ten main medicolegal issues (quality, patient satisfaction, safety, risk, privacy, practitioner satisfaction, ethics, misdiagnosis, informed consent, and medical liability). The selection of the relevant issues was based on the authors’ opinions summarized with the estimate-talk-estimate Delphi method, which consists of an open opinion exchange workshop. The authors verified whether the relevant medicolegal issues were considered or not in the selected records and expressed such measures as a proportion. The distribution of positive/negative outcomes was tested using the chi-square test, splitting the records according to the explored medical field of intervention (diagnostics, therapy, follow-up, unspecified). The *p*-value was considered significant when *p* < 0.05.

Data were collected with Microsoft Excel and analyzed with STATA.

## 3. Results

**Figure 1:**
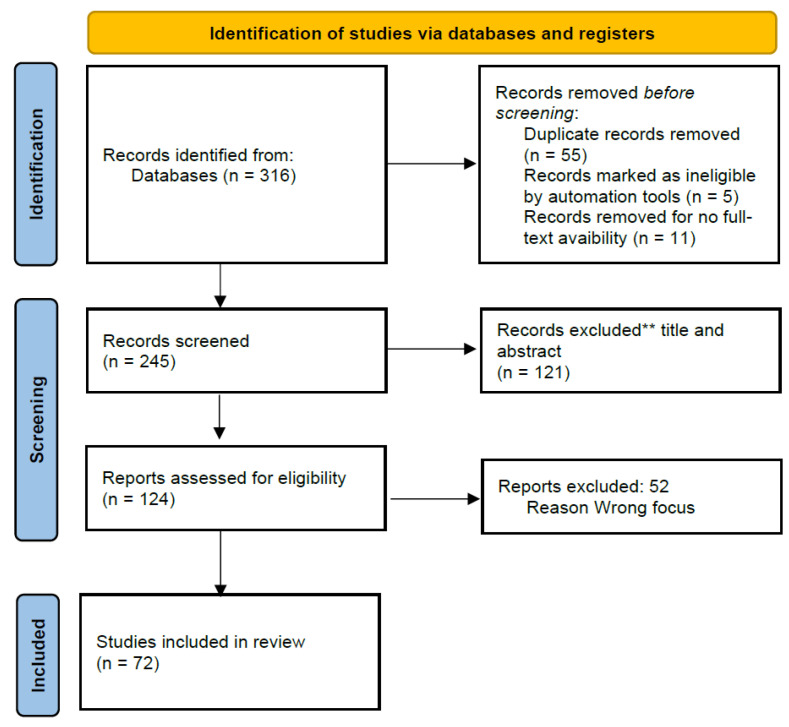
Flow diagram describing the review process, according to PRISMA statement.

The searches returned 316 results, 55 of which were duplicates, five were excluded due to ineligibility by automation tools, and 11 were excluded because no full text was available. The screening process excluded 121 papers, while the full-text assessment excluded 52 reports. Finally, the elected records were 72. Figure 1 illustrates the flow diagram describing the review process (Figure 1).

The papers were published from 2017 to 2021 with the following distribution: One in 2017 (1.4%), four in 2018 (5.6%), seven in 2019 (9.7%), 22 in 2020 (30.6%), and 38 in 2021 (52.8%). The annual percentage increase in the number of publications was 132.4% (214.3% between 2019 and 2020).

The authors’ institutions were based in the USA (37 papers, 51.4%), UE (16 papers, 22.2%), UK (nine papers, 12.5%), Canada (six papers, 8.3%), Qatar (one paper, 1,4%), China (one paper, 1.4%), Republic of Korea (one paper, 1.4%), and Brazil (one paper, 1.4%). No records were issued for telesurgery.

The number of original research papers was 44 (61.1%), 20 records were reviews (27.8%), and eight records (11.1%) were other types of papers (letters to the editor, commentary, others). Thirty-seven original research papers directly compared tele-orthopedics and face-to-face orthopedics (84.0%).

The valued medical activity was a follow-up in 23 papers (31.9%), therapy in 14 cases (19.4%), and diagnostics in 14 cases (19.4%), meanwhile, 21 records were not so specific (29.2%).

In the 37 original research papers, tele-orthopedics was compared to face-to-face orthopedics. Among such papers, 34 expressed a clear preference for tele-orthopedics (91.9%), and three considered that face-to-face orthopedics provided better results (8.1%). Table 1 shows how the positive main outcome changed among the considered medical fields of intervention with no significant differences (*p* > 0.05) (Table 1). Both the positive and negative main outcomes demonstrated no significant association with the medical fields of interventions (diagnostics, therapy, follow-up, undefined) described in the record (*p* > 0.05).

The selected issues were assessed with the following distribution (Table 2).

## 4. Discussion

This review showed that the articles published on tele-orthopedics topics had prevailing areas of interest related to legal medicine and clinical risk management. All the selected records were qualitative research, so it was not possible to perform a meta-analysis.

The authors selected and reviewed 72 papers, 60 of which were published in 2020 (22) and 2021 (38). Hence, the interest in tele-orthopedics received a strong push from the COVID-19 pandemic. The pandemic has indeed forced orthopedic practitioners to adapt, quickly hastening the implementation of telemedicine [13]. According to Moisan et al., telemedicine can increase productivity and access to orthopedic care [14].

In 46 cases, the authors’ institutions were based in North America (the USA and Canada), i25 cases in Europe (UE and UK), three cases in Asia (China, Republic of Korea, and Qatar), and one case in South America (Brazil). Despite several authors stating that telemedicine may overcome geographical barriers to health care, the scientific interest in tele-orthopedics seems to involve only a few countries. According to Krus et al., telemedicine (including tele-orthopedy) is not yet ubiquitous, and several barriers still limit its spread [15]. The access to technological aids indeed varies widely according to the considered country. Moreover, the acceptance of new technologies lies in cultural aspects (e.g., perceived ease of use, perceived usefulness, trust in personal data management, and others). The above raises an important aspect of sensitizing telemedicine professionals and their users to cultural issues where the adoption of standard definitions and protocols could help overcome these issues [16,17,18,19,20,21,22]. Notwithstanding that in 2002, Loane raised concerns regarding the scarcity of guidelines and standards in telemedicine, the selected studies did not assess the issue [23].

Among the selected papers, 44 were original research papers (61.1%), 20 were reviews (27.8%), and eight (11.1%) were other types of papers. Among the original research articles, 37 papers included a comparison between tele-orthopedics and face-to-face orthopedics. The authors summarized the qualitative results of such a comparison by assigning a “positive” or “negative” main outcome. The main outcome was considered positive when the research stated tele-orthopedics as a feasible alternative to face-to-face orthopedics according to its main outcomes. Only three records (8.1%) reported a negative main outcome, with no significant difference among the main clinical activity (diagnostics, therapy, follow-up, undefined), while 34 records (91.9%) reported a positive main outcome. Hence, tele-orthopedics is widely considered at least as good as face-to-face orthopedics concerning medicolegal and risk management issues. On the other hand, due to the number and the general quality of primary studies concerning the medicolegal aspects of tele-orthopedics, strong evidence in the field still seems far from being produced. All of the selected records were qualitative studies performed by surveys, which entails poor results in terms of evidence strength. Moreover, the enthusiastic point of view of many authors may suffer from positive-publication bias [24].

The authors selected several medicolegal and clinical governance issues that can be related to telemedicine and tele-orthopedics. Quality was the most common topic (83.3%) of the reviewed articles. In 77.8% of the records, issues related to patient satisfaction were analyzed, while in 51.4%, issues related to the concept of safety were considered. These data show that the spread of clinical risk management is now a reality as the introduction of ICT in medicine evaluated the quality of care, patient satisfaction, and safety. This, undoubtedly, is an extremely positive aspect that demonstrates that the health professional–patient therapeutic alliance is seen as the main goal to be pursued in treatment through the spread of a no-blame culture [25]. Hence, it can be confirmed that the authors who dealt with tele-orthopedics focused mainly on systemic clinical risk management as a tool to improve the quality of care and patient safety [26].

On one hand, issues related to legal medicine have been less considered despite the sensitivity of the issues. Only 30.6% of the reviewed articles analyzed the ethical issue, while the issues related to misdiagnosis, informed consent, and medical liability were the object of attention in 23.6%, 15.3%, and 13.9% of the reviewed articles, respectively. These data led to careful reflection, as while discussing tele-orthopedics, its main medicolegal pitfalls were substantially neglected. Nevertheless, using technology and aids, telemedicine has the potential to enhance patient understanding and improve the informed consent acquisition process [27,28].

In terms of professional liability, telemedicine services are comparable to any diagnostic-therapeutic health service considering that it does not replace the traditional health service but integrates the latter to improve its effectiveness, efficiency, and appropriateness. Hence, the health service mediated by ICT is normally considered equal to the service provided in traditional forms [29,30]. Despite the importance of the doctor-–patient relationship, malpractice, standardization of adopted practices, and economic reimbursement in telemedicine [31], these issues have not yet been received and analyzed by the community that deals with tele-orthopedics. Moreover, telemedicine and tele-orthopedics entail specific risks originating from equipment defects, system failures, ineffective maintenance, inadequate management, and the incorrect transmission and evaluation of data. This may lead to medical professional liability claims, so much so that some services necessarily require the physical presence of the physician or his virtual participation [32]. Given such context, professional liability in tele-orthopedics seems to deserve specific attention. Moreover, telemedicine has the potential to allow for cross-national health care, with rising concerns about the different regulations regarding professional claims.

The misdiagnosis is issued by only 26.3% of the selected studies. Given the increase in teleconsultation, the lack of interest in this field deserves a specific mention [33,34].

The privacy issue appears to be quite neglected, even if the spread of telemedicine and the digitalization of health care may allow for the collection of private data and their secondary use. However, the latest studies in the field have proposed different tools or architectures to protect personal health data, which may be largely diffused in the future [35,36].

The results of this review confirm that even the use of tele-orthopedics, if also properly analyzed from the point of view of medicolegal issues, can have a positive impact on health systems in terms of the involvement of the operators within the care and decision-making processes, implementation of the quality and safety levels of the treatments, and a reduction in litigation with a view of modern medicine that is less and less “defensive” and more and more “no blame” [37]. Undoubtedly, telemedicine in orthopedics has the potential to provide high-quality orthopedics services to patients in remote areas with the recent release of telemonitoring (teleconsultation and telemetry) and telesurgery (consisting of the use of wireless networking and robotic technology to operate on patients who are distantly located), which are the two major forms of tele-orthopedics [38,39]. However, the selected papers mainly concerned orthopedic follow-up, as teleconsultation during post-surgical rehabilitation seemed to be the most explored issue.

Despite this, tele-orthopedics was found to be disruptive as it requires the redesign of many care processes [40,41,42,43,44]. Moreover, the absence of strong evidence on the clinical and medicolegal problems that the use of telemedicine can determine represents a weakness while spreading tele-orthopedics [40,41,42,43,44].

Limitations include the lack of telehealth utilization in many countries and that most reports are descriptive implementation studies. Another limitation is the lack of metanalysis due to the absence of quantitative evidence.

More research addressing the risks and benefits of telehealth is urged, considering the rapid spread of telemedicine and the increasing digitalization of clinical research. 

## 5. Conclusions

The analyzed literature showed a large increase in scientific publications regarding tele-orthopedics in the last two years, related to the COVID-19 pandemic. The pandemic emergency and the consequent increase in telemedicine services allowed us to examine the relevant ethical and legal issues and to study the fundamental responsibilities during the period of a health emergency. Telemedicine programs improve access and have redefined the platforms in which patients receive medical treatments, physicians deliver medical treatments, and the relationships that are formed between these two parties. 

The spread of telemedicine and the increase in the digitalization of health care pose potential medicolegal concerns, related to medical liability but also to privacy protection, the quality and safety of health care, and patient/provider satisfaction. The development of standards for telemedicine practice and effectiveness studies are recommended.

## Figures and Tables

**Table 1:** The main outcomes of the different medical fields of intervention among the 37 records that compared tele-orthopedics and face-to-face orthopedics.

Main Outcome	The Medical Field of Intervention			
	Diagnostics	Therapy	Follow-Up	Undefined
Positive	9	4	14	7
Negative	0	2	1	0

**Table 2:** Medicolegal and risk management problems among the 72 selected records.

Issues	Considered (%)	Not Considered (%)
Quality	60 (83.3)	12 (16.7)
Patient Satisfaction	56 (77.8.)	16 (22.2)
Safety	37 (51.4)	35 (48.6)
Risk	29 (40.3)	43 (59.7)
Privacy	26 (36.1)	46 (63.9)
Practitioner satisfaction	23 (31.9)	49 (68.1)
Ethics	22 (30.6))	50 (69.4)
Misdiagnosis	17 (23.6)	55 (76.4)
Informed consent	11 (15.3)	61 (84.7)
Medical liability	10 (13.9)	62 (86.1)
